# *QuickStats*: Percentage* of Uninsured Adults Aged 18−64 Years,^†^ by Race, Hispanic Origin, and Selected Asian^§^ Subgroups — National Health Interview Survey, United States, 2019−2020

**DOI:** 10.15585/mmwr.mm7128a5

**Published:** 2022-07-15

**Figure:**
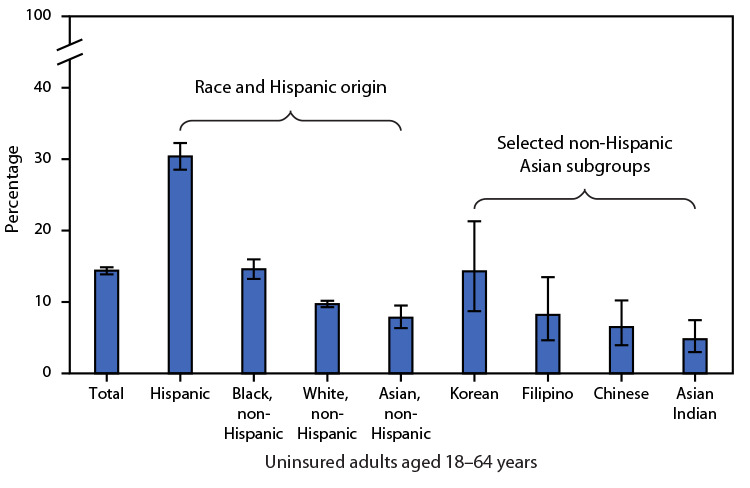
During 2019–2020, the percentage of U.S. adults aged 18–64 years who were uninsured was 14.4%. Among all race and Hispanic origin groups, non-Hispanic Asian adults (7.8%) were the least likely to be uninsured followed by non-Hispanic White (9.7%), non-Hispanic Black (14.6%), and Hispanic adults (30.4%). Among the non-Hispanic Asian subgroups shown, adults of Korean (14.3%) origin were more likely to be uninsured than adults of Asian Indian (4.8%) and Chinese (6.5%) origin. Other observed differences were not statistically significant.

**Source:** National Health Interview Survey, 2019 and 2020 data. https://www.cdc.gov/nchs/nhis.htm

